# Knowledge, awareness, and perception of dental students, interns, and freshly graduated dentists regarding dental implant complications in Saudi Arabia: a web-based anonymous survey

**DOI:** 10.1186/s12903-021-01506-2

**Published:** 2021-03-25

**Authors:** Islam Saad, Suzan Salem

**Affiliations:** 1 Department of Periodontology and Oral Medicine, College of Dentistry, Qassim University, King Abdulaziz Road, Mulaidah, Buraidah, Qassim 51452 Kingdom of Saudi Arabia; 2 Department of Maxillofacial Surgery and Diagnostic Science, College of Dentistry, Qassim University, King Abdulaziz Road, Mulaidah, Buraidah, Qassim 51452 Kingdom of Saudi Arabia

**Keywords:** Knowledge, Awareness, Perception, Dental students, Dental implant complications, Saudi Arabia

## Abstract

**Background:**

It is necessary for dental students and freshly graduated dentists to apply their education and practice to different clinical and preclinical procedures. The implant success rate and durability are high. Therefore, this study was designed to assess the level of knowledge, awareness and perception of dental students, interns and freshly graduated students regarding dental implant complications in Saudi Arabia.

**Methods:**

A cross-sectional study design using a web-based method was conducted at different dental institutions in Saudi Arabia from December 2019 to March 2020. Data were collected from all (n = 288) undergraduate students, interns and freshly graduated dentists using a pretested standardized self-administered web-based questionnaire that was prepared and circulated using a template provided by Google Forms (Google, Inc., USA). Descriptive statistics and chi-square tests were performed to analyse the data using SPSS (version 20).

**Results:**

A total of 288 participants completed the questionnaire, with a response rate of 83.4%. Of the total participants, 37.5% showed a good level of knowledge regarding dental implant procedures, and 38.9% showed the same level of knowledge regarding implant complications. The most important cause of complications associated with dental implants was case selection, which accounted for 54.17%. Most participants (58.33%) chose massive bone loss related to implant failure as the most common late dental complication, while 26.39% chose postoperative infection as the most common early dental complication. In addition, 30.56% of the participants selected screw loosening as the most frequent mechanical complication. However, lack of implant primary stability was the most common hard-tissue implant complication. Based on aesthetic and reversible complications, restorations that were too buccal or too palatal and prosthetic-related, respectively, were the least common dental implant complications.

**Conclusions:**

The level of knowledge among participants regarding dental implant complications differed among the institutions participating in this study. This difference reflects a significant variation that necessitates reviewing and standardizing dental implant curricula among these institutions.

**Supplementary Information:**

The online version contains supplementary material available at 10.1186/s12903-021-01506-2.

## Background

Modern dentistry aims to restore the patient to normal function, aesthetics, speech, and health. The ability to achieve these ideal objectives with implant dentistry is exceptional and because of its effectiveness and predictability, the use of dental implants in the rehabilitation of partially and fully edentulous jaws has become a well-established and accepted modern therapeutic procedure [1–3].

However, complications can occur in the process or as an outcome of treatment, which does not allow patients to benefit entirely from the intended therapeutic interventions. Most medical errors and complications are believed to be preventable; therefore, extensive research, educational programmes, and government policies are geared towards complication prevention [4].

Although the medical literature tends to use several terms to refer to adverse problems or their risks, "complication" is still the most widely used term in the literature on dental implants. Using the word “complication” does not automatically imply inaccuracy during treatment planning, execution, or follow-up or a direct negative impact on the patient [5]. The skilful efforts of dental team members (including laboratory technicians) play an essential role in preventing these negative impacts on the patient, as does the consistent willingness of the patient to adapt to or acknowledge minor deviations from ideal aesthetic appearance, shape or function [6–8].

No single, universally accepted classification system for implant-related complications exists. Several approaches to classifying all or some implant complications have been suggested. In classifying implant complications, authors have established two general approaches: some authors have attempted to classify all types of implant complications [8, 9], while other authors have attempted to classify only some implant complications, united either by the particular phase of therapy during which they tend to occur (such as surgical [9, 10] or prosthodontic complications [9, 11]) or by some other feature in the process (e.g., reversible complications [12]) or outcome of care (e.g., aesthetic complications [13]).

The vast majority of data regarding these complications come from universities. Large university-based research centres (e.g., Gothenburg, Leuven, Malmo, Mayo, and Toronto) represent the overwhelming majority of published implant treatment results [9, 14]. Several studies have raised the issue of insufficient dental implant education at the undergraduate level [15–18].

In Saudi Arabia and elsewhere, several studies regarding the practice of implant dentistry refer to practitioners’ level of education. Education and training in implant dentistry in different countries can also vary, including undergraduate and formal postgraduate training, fellowship/board training and the attendance of courses and/or seminars [19–21]. Most previous studies have been designed to measure the level of awareness and knowledge regarding dental implants among undergraduate students, among patients or among general practitioners. One of these studies, which was conducted in Saudi Arabia, showed that for a better understanding of dental implants, the complications and management of undergraduate dental implant programs should be modified to provide better care for patients [20].

While different types of implant complications that can be encountered are well known, the level of knowledge and awareness of these complications among undergraduate and graduate students in Saudi Arabia is still unknown. This study was designed to determine the level of knowledge and the subjective and objective need for information about all types of dental implant complications among students, interns, and freshly graduated dentists of different institutions in Saudi Arabia.

## Methods

### Study design and period

A cross‑sectional web‑based questionnaire‑based study was conducted from December 2019 to March 2020. The focus of the study was undergraduate students, interns and freshly graduated dentists from different dental institutions in Saudi Arabia. This study was approved by the Research Ethics Committee of the College of Dentistry Qassim University, ref no. ST/6064/2019.

### Sample size determination

Participants were selected using multistage cluster sampling in which the twenty institutions were divided into four clusters according to their geographic distribution. From each cluster, individual units were selected randomly for use as samples. Calculating the sample size for the given sampling frame using a 95% confidence level (α = 0.05), 5% confidence interval and statistical power of 0.85, a study sample of 278 participants (n = 278) was required to achieve a statistically valid result.

### Quantitative data collection tools and techniques

Quantitative data were collected using a template provided by Google Forms (Google, Inc., USA). The setting of response was set to be one response to prevent multiple entries from the same participant. The study protocol was explained to all participants who participated in the study, and written informed consent was obtained prior to completion of the questionnaire. A self-explanatory English-language closed‑ended questionnaire was designed by the authors based on data reported previously in the literature [9–13, 22]. The list of variables included knowledge and awareness about dental implants and their complications, factors responsible for complications associated with dental implants, early and delayed complications associated with dental implants, mechanical complications associated with dental implants, soft and hard tissue complications associated with dental implants and, finally, aesthetic and reversible complications associated with dental implants.

### Validity and reliability

A questionnaire was developed and composed of three parts. The first part included the demographic data of the participants. The second part aimed to assess the knowledge and awareness of the participants regarding dental implants and their associated complications. The third part aimed to assess the knowledge and awareness of the participants regarding different types of dental implant complications. The questionnaire was reviewed by a panel of experts in dental implantology at the College of Dentistry, Qassim University. This process verified the content validity of the questionnaire. Cronbach’s alpha was found to be 0.847, which is good for a new questionnaire (Additional file 1).

### Pilot study

The questionnaire was tested by distributing it to 50 dental students and interns drawn from the same sampling frame who filled out the same form 1 month prior to conducting the study to evaluate the applicability, ease of understanding and clarity of the questions. The feedback obtained from the pilot survey was used to refine the questionnaire and to simplify questions that were not easily understood.

### Data analysis

The data were coded, tabulated, and analysed using the Statistical Package for Social Sciences software (IBM SPSS, Inc., Chicago, version 20) (Additional file 2). A chi-square test was used to determine whether there was any statistically significant difference in the participants’ knowledge and attitudes based on gender. A *p* value of less than or equal to 0.05 was considered statistically significant throughout the analyses (Additional file 3).

## Results

A total of 288 responses were obtained from dental students, interns and freshly graduated dentists across the country, with a response rate of 83.4%. The response rate was higher than the sample size calculated to achieve significant valid results. Of the total number of participants, 200 (69.44%) were males and 88 (30.56%) were females. They were distributed among seven institutions in Saudi Arabia at different educational levels (Table 1).

**Table 1:** Frequency regarding gender, education level and institution (n = 288)

Frequency of demographic characteristics		
	Frequency	%
*Gender*		
Female	88	30.56
Male	200	69.44
*Education level*		
4th year students	24	8.33
5th year students	92	31.94
Intern	100	34.70
Freshly graduates	72	25.0
*Institution*		
King Abdulaziz University	26	9.03
King Faisal University	22	7.64
King Khalid University	57	19.79
Qassim University—Arras	36	12.50
Qassim University—Al-Mulidah	69	23.96
Taibah University	44	15.28
Umm Al-Qura University	34	11.81

**Fig. 1:**
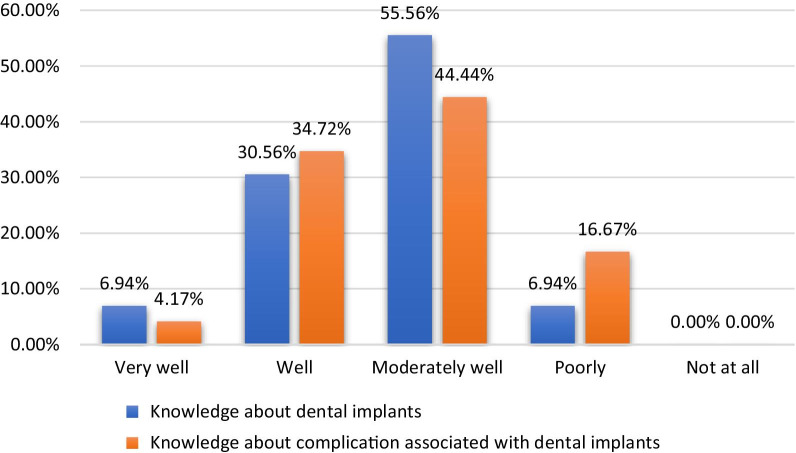
Percentage of participants with knowledge about dental implants and the associated complications

The majority 55.56% (n = 160) of participants were moderately knowledgeable, whereas 37.5% (n = 108) had good knowledge about dental implant procedures. Moreover, significantly higher (*p* < 0.001) differences in knowledge about dental implants were observed among the institutions and educational levels. However, no significant association between the knowledge and gender of the participants was found (Table 2 and Fig. 1).

**Table 2:** Differences among participants regarding institution, education level and gender (n = 288)

		University	Education level	Gender
Knowledge about dental implants	*χ*^2^	51.439	53.158	3.994
	*p*	*p* < 0.001*	*p* < 0.001*	0.262
Awareness of complications associated with dental implants	*Χ*^2^	32.306	118.699	13.806
	*p*	0.020*	*p* < 0.001*	0.003*
Factors responsible for dental implant complications	*χ*^2^	42.06	84.292	32.836
	*p*	0.071	*p* < 0.001*	*p* < 0.001*
Early complications associated with dental implants	*χ*^2^	50.696	86.416	8.537
	*p*	0.010*	*p* < 0.001*	0.129
Late complications associated with dental implants	*χ*^2^	29.045	48.726	6.45
	*p*	0.048*	*p* < 0.001*	0.092
Mechanical complications associated with dental implants	*χ*^2^	51.078	79.612	26.61
	*p*	0.049*	*p* < 0.001*	*p* < 0.001*
Soft tissue complications associated with dental implants	*χ*^2^	56.956	47.037	27.316
	*p*	0.002*	*p* < 0.001*	*p* < 0.001*
Hard tissue complications associated with dental implants	*χ*^2^	38.24	109.423	22.826
	*p*	0.144	*p* < 0.001*	*p* < 0.001*
Aesthetic complications associated with dental implants	*χ*^2^	42.742	44.555	51.062
	*p*	0.062	*p* < 0.001*	*p* < 0.001*
Reversible complications associated with dental implants	*χ*^2^	61.968	78.718	20.87
	*p*	0.001*	*p* < 0.001*	0.001*

*The chi-square statistic is significant at the 0.05 level

The majority 44.44% (n = 128) of participants were moderately knowledgeable, whereas 38.89% (n = 112) had good knowledge about complications associated with dental implants. Moreover, significantly higher (*p* < 0.001) differences in knowledge about complications associated with dental implants were observed among the educational levels. However, the differences among participants from different institutions and between the genders were statistically significant (*p* < 0.05) (Table 2 and Fig. 1).

**Fig. 2:**
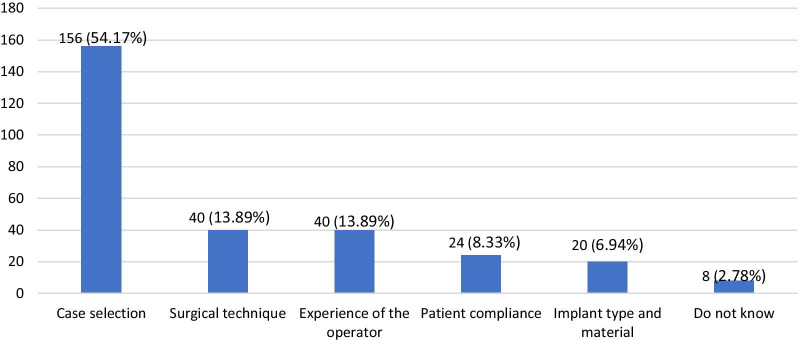
Percentage of participants with knowledge about the most important factor responsible for dental implant complications

Most participants 54.17% (n = 156) identified case selection as the most important factor responsible for complications associated with dental implants, whereas patient compliance, surgical technique, implant type and material, or experience of the operator were identified as the least important factors responsible for implant complications. Moreover, significantly higher (*p* < 0.001) differences regarding factors responsible for dental implant complications were observed among participants at different educational levels and between genders. However, no significant difference between factors responsible for dental implant complications among the different institutions was found (Table 2 and Fig. 2).

**Fig. 3:**
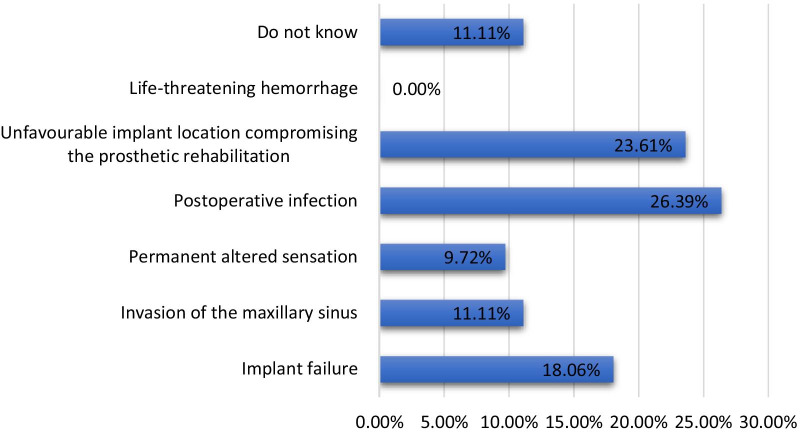
Percentage of participants with knowledge about early complications associated with dental implants

**Fig. 4:**
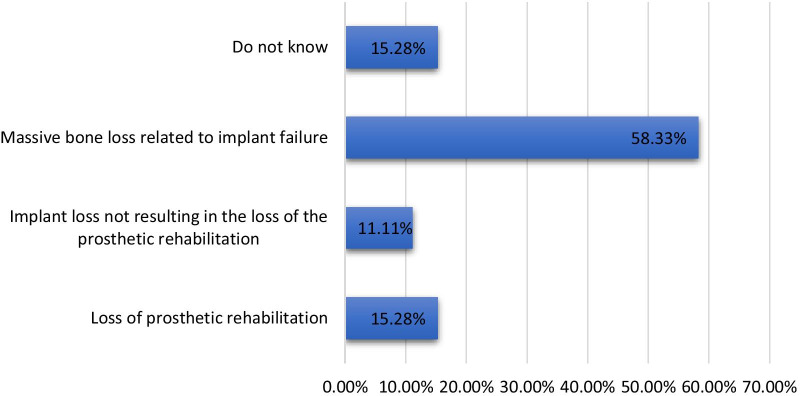
Percentage of participants with knowledge about late complications associated with dental implants

Of the total participants, 26.37% (n = 76) and 23.61% (n = 68) reported that postoperative infection and unfavourable implant location compromising prosthetic rehabilitation were the most common early complications associated with dental implants, respectively, whereas 11.11% (n = 32) reported a lack of knowledge regarding early complications. On the other hand, the majority of the participants 58.33% (n = 168) identified massive bone loss related to implant failure as the most common late complication associated with dental implants, whereas 15.28% (n = 42) reported a lack of knowledge regarding late complications (Figs. 3, 4).

Moreover, significantly higher (*p* < 0.001) differences regarding early and late complications associated with dental implants were observed among participants at different educational levels. However, the differences among participants from different institutions were statistically significant (*p* < 0.05), and no significant difference between gender was found (Table 2).

**Fig. 5:**
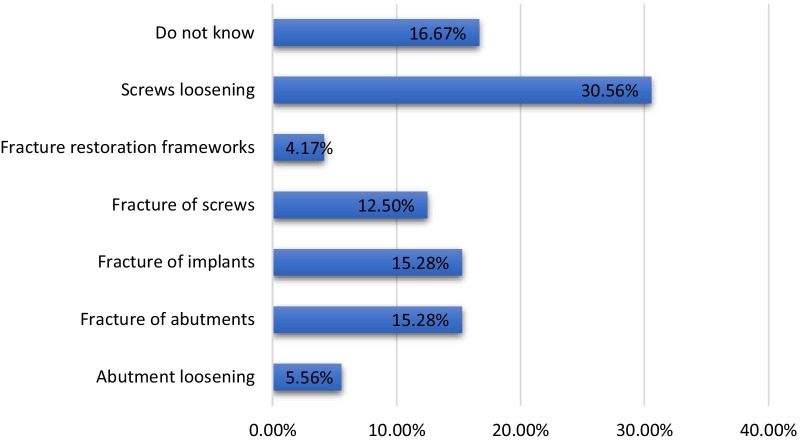
Percentage of participants with knowledge of mechanical complications associated with dental implants

As many as 30.56% (n = 88) of participants identified screw loosening as the most common mechanical complication, whereas abutment loosening and fracture restoration frameworks were selected as the least common mechanical complications associated with dental implants; 16.67% (n = 48) of participants showed a lack of sufficient information regarding this complication. Moreover, significantly higher (*p* < 0.001) differences in knowledge about mechanical complications were observed among participants at different educational levels and between genders. However, the differences among participants from different institutions were statistically significant (*p* < 0.05) (Table 2, Fig. 5).

**Fig. 6:**
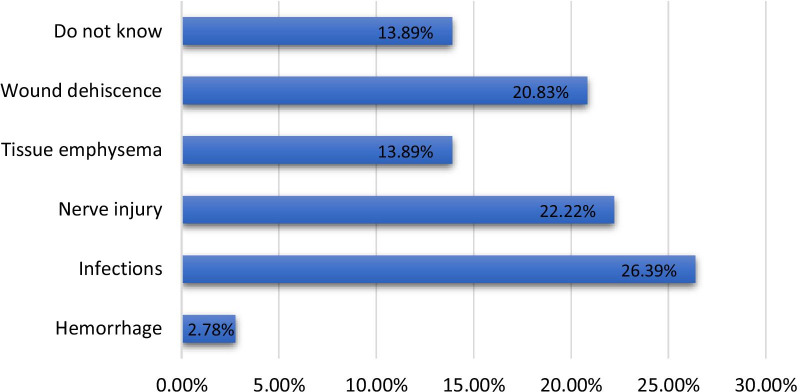
Percentage of participants with knowledge of soft tissue complications associated with dental implants

Of the total participants, 26.39% (n = 76), 22.22% (n = 64) and 20.83% (n = 60) responded similarly that infection, nerve injury and wound dehiscence, respectively, were the most common soft tissue complications associated with dental implants. Moreover, significantly higher (*p* < 0.001) differences in knowledge about soft tissue complications associated with dental implants were observed among participants from different institutions, at different educational levels and between genders (Table 2, Fig. 6).

**Fig. 7:**
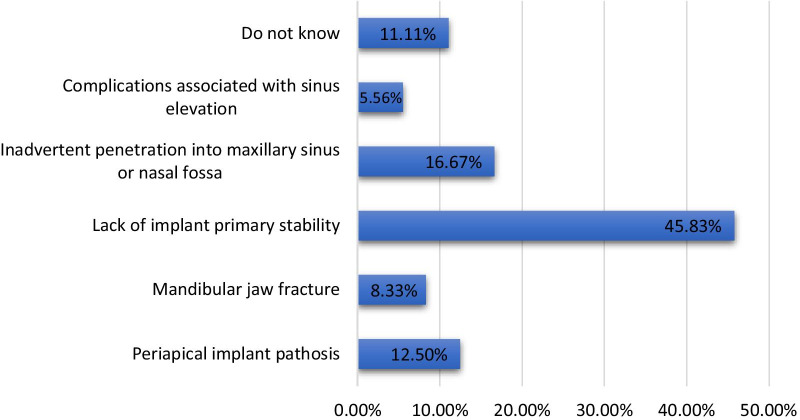
Percentage of participants with knowledge of hard tissue complications associated with dental implants

On the other hand, the majority of the participants (45.83%, n = 132) agreed that a lack of implant stability was the most common hard tissue complication associated with dental implants, whereas 11.11% (n = 32) reported a lack of knowledge regarding these complications. Moreover, significantly higher (*p* < 0.001) differences in hard tissue complications associated with dental implants were observed among participants at different educational levels and between genders. However, no significant difference was found between the knowledge and gender of the participants (Table 2, Fig. 7).

**Fig. 8:**
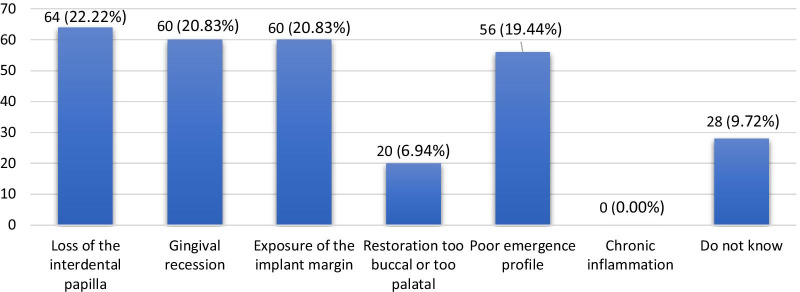
Percentage of participants with knowledge of aesthetic complications associated with dental implants

Regarding knowledge of aesthetic complications associated with dental implants, 22.22% (n = 64), 20.83% (n = 60), 20.83% (n = 60) and 19.44% (n = 56) of participants responded similarly that loss of the interdental papilla, gingival recession, exposure of the implant margin and poor emergence, respectively, were the most common aesthetic complications. Moreover, significantly higher (*p* < 0.001) differences in knowledge about aesthetic complications associated with dental implants were observed among participants at different educational levels and between genders. No significant differences were found in knowledge among participants from different institutions (Table 2, Fig. 8).

**Fig. 9:**
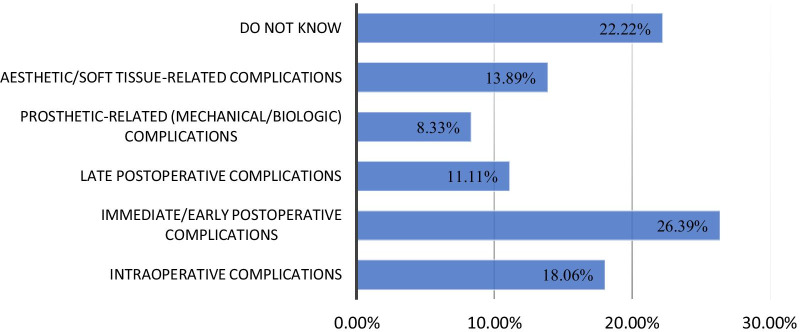
Percentage of participants with knowledge of reversible complications associated with dental implants

Of the total participants, 26.39% (n = 76) answered that immediate/early postoperative complications were the most common reversible complications associated with dental implants, whereas 22.22% (n = 64) expressed poor knowledge regarding these complications. Moreover, significantly higher (*p* < 0.001) differences in knowledge about reversible complications associated with dental implants were observed among participants from different institutions, at different educational levels and between the genders (Table 2, Fig. 9).

## Discussion

The survey was conducted with a representative population of fourth- and fifth-year students, interns and freshly graduated dentists at different dental institutions in Saudi Arabia. This population represents the future of dentistry; therefore, it is important to assess their knowledge and attitudes towards implant dentistry.

Despite the high survival rate of dental implants, several systematic reviews have attempted to identify and quantify the occurrence of complications related to treatment with endosseous dental implants. The most comprehensive reviews have examined the entire scope of complications, from the surgical appointment to the latest follow-up [22, 23]. Other reviews have been limited to specific phases of therapy, such as surgical or immediate postoperative complications [10, 24] or complications that might occur over the maintenance period [25–30].

In Saudi Arabia, most dental schools offer equal education for male and female students. However, in the current study, males were represented more predominately than their counterparts. The predominance of males over females was also reported in a previous study, which attributed this finding to other factors, stating that the dental profession worldwide is generally more male dominated [31]. However, this finding is inconsistent with other studies [32–35]. One of these studies, which was conducted in India, attributed this shift to the growing increase in females over males that has occurred over several years [33]. Whether this gender predominance may be attributed to desire remains to be evaluated.

Most participants were in their 5th year, and interns were among the responding participants at different academic levels, though not at the expected proportions. A higher number of the most appropriate evidence-based answers was expected from freshly graduated dentists. These results reflect the level of education of 5th-year students and interns over 4th-year students but do not provide an indication of freshly graduated dentists’ knowledge. Sharma and his colleagues also found a higher number of female students in the 5th year and 4th year among the students who participated in their survey. Moreover, the researchers noted that even in the late-clinical year, a majority of students gave unsatisfactory answers [35].

The role of dental education and modern dental curricula in providing dental implant education has been extensively studied [16–18]. Similar to international programmes, dental schools in Saudi Arabia offer a 1-year mandatory clinical training programme before graduation [36]. The results of the current study showed a significant difference and sometimes highly significant differences among different dental institutions in Saudi Arabia, which should be re-evaluated to standardize the learning outcome in relation to didactic and practical courses on dental implants. In 2019, Albugami conducted a study to assess the education and training of dentists practising implantation and concluded that advanced clinical specialist training will inevitably lead to an improvement in the quality of implant therapy for the benefit of patients [21].

Most of the participants had moderate knowledge regarding dental implants. This finding is similar to the corresponding result of a previous study performed in India [37]. In addition, most of the participants reported being “moderately well informed” about and aware of complications associated with dental implants. Several observations suggest that numerous complications are encountered in clinical practice; however, severe complications are relatively rare [38]. Most of the specific complications reported in these studies by a large proportion of the survey participants are relatively easily managed and largely without permanent detrimental effects [9, 11, 22, 23].

Most of the participants agreed that the most important factor for implant success is case selection. Patient selection represents a priority when dental implants are being considered. History taking and examination are very important to assess the patient’s willingness and compliance to undergo surgery [39–41]. Similar results were obtained from previous studies conducted in Saudi Arabia as well as in Sweden, which identified patient noncompliance and poor oral hygiene as the main reasons underlying these clinical complications [20, 42].

A large number of endogenous and exogenous factors have been identified as causative or contributory factors to implant failure [3, 43, 44], and several theories have been advanced to explain the mechanisms of implant failure [45]. Most theories focus on infectious agents, healing deficits and loading factors to account for the clustering of implant failures soon after implant placement (early failure) or within 1 year of loading (late failure). In our study, only 26.37% of participants reported that postoperative infection was the most common early complication, while 23.61% agreed that an unfavourable implant location compromising prosthetic rehabilitation was the most common early complication. The majority of the participants identified massive bone loss related to implant failure as the most common late complication associated with dental implants.

In 2020, a study was conducted to evaluate the effect of dynamic cyclic loading on screw loosening in both narrow and standard implants and concluded that screw loosening occurred in both narrow and standard implants, with a higher value in narrow implants [46]. Similar results were found in a previous systematic review of single implant-supported restorations, which concluded that screw loosening occurred with a cumulative incidence of 12.7% after 5 years, while loss of retention due to fracture of luting cement occurred with a cumulative incidence of 5.5% after 5 years [9]. These results are similar to those of our investigation. However, another study on this topic concluded that abutment screw loosening in single-implant restoration is a rare event regardless of the geometry of the implant-abutment connection (external or internal) if proper anti-rotational features and torque are employed [28].

Our results showed that a lack of primary stability is the most common hard tissue complication associated with dental implants. These results were in accordance with studies conducted by Duyck and Esposito, who reported that primary stability is an important determinant of future implant success [43, 44] and is a key determinant in advanced implant treatment approaches such as immediate placement and immediate loading [47].

Although the importance of peri-implant inflammation and infection as a significant cause of implant failures is controversial and may not be applicable to all implant systems [44], 26.39% of the participants considered infection to be the most common soft tissue complication associated with dental implants. Several soft tissue complications that are difficult to manage or are likely to result in significant patient disability (such as nerve injury, haemorrhage and wound dehiscence) were reported by a lower percentage of the participants.

In our study, participants responded similarly that loss of the interdental papilla, gingival recession, exposure of the implant margin and a poor emergence profile are the most common aesthetic complications associated with dental implants. Recent studies in the literature have reported aesthetic complications, revealing that between 4 and 16% of single implant crowns in the anterior maxilla fail for aesthetic reasons [48–50]. The most common aesthetic complication is gingival recession exposing the implant/abutment junction, with one study reporting up to 61% of cases with at least 1 mm of gingival recession on the facial aspect. Poor shade selection for the prosthesis and lack of interdental papillae also account for implant aesthetic failures [51, 52].

Reversible complications may occur at various phases of procedures, including intraoperatively, early/late postoperatively, and during prosthetic reconstruction and/or after functional loading. In 2005, Park and Wang presented their accepted classifications and treatments of various reversible complications commonly encountered during routine implant-related procedures [12]. Several studies were conducted based on Park and Wang’s classification to evaluate these complications; it was concluded that careful clinical and radiographic examination of each case, accurate planning of procedures, the use of proper surgical techniques and appropriate instruments, and the correct management of healing and osseointegration all combine to prevent such events [53–57]. In our study, 22.22% of participants expressed poor knowledge regarding these complications, which may be explained by the unfamiliarity of this type of reversible complication classification among the participants.

Several limitations were encountered in this study. A new questionnaire was introduced, and most of the surveyed participants were undergraduates and fresh graduates, which must be taken into account when evaluating the findings. A further survey that includes more detailed questionnaires and a greater number of institutions and participants, particularly freshly graduated dentists, is recommended to validate the results of the present study.

## Conclusion

The present study indicates differences in the knowledge and perceptions of the complications of dental implants among dental students and freshly graduated dentists from different institutions in Saudi Arabia. The participants expressed a lack of knowledge regarding reversible complications. In addition, they demonstrated similar responses regarding aesthetic and soft tissue complications. Hence, the present study concluded that participants showed significant variation that necessitates reviewing and standardizing dental implant curricula among different institutions. This investigation therefore suggests a need for curriculum review, evaluations of teaching materials and methods, consensus workshops and hands-on training in undergraduate studies that would result in a better understanding of dental implants and their complications so that students can respond properly to the increasing number of patients with queries about dental implants.

## Supplementary Information

**Additional file 1.** The questionnaire distributed among participants.

**Additional file 2.** The raw data collected from the questionnaire.

**Additional file 3.** Chi square of the data collected data.

## Data Availability

All data generated or analysed during this study are included in this published article [and its supplementary information files].
