# Supplementary material for: Knowledge, awareness, and perception of dental students, interns, and freshly graduated dentists regarding dental implant complications in Saudi Arabia: a web-based anonymous survey
Source: BMC Oral Health. 2021 Mar 25;21:161. doi: 10.1186/s12903-021-01506-2 (PMC7995726; doi:10.1186/s12903-021-01506-2)
Supplement: Supplementary file 1 — Additional file 1. The questionnaire distributed among participants. [file 12903_2021_1506_MOESM1_ESM.pdf]

1. **How satisfactory are you regarding the knowledge about dental implant?**
   a. Very well
   b. Well
   c. Moderately well
   d. Poorly
   e. Not at all

2. **How much are you aware of complication associated with dental implants**

a. Very well
b. Well
c. Moderately well
d. Poorly
e. Not at all

3. **What do you think is the most important factor responsible for complication associated with dental implant?**

a. Case selection
b. Implant type and material
c. Patient compliance
d. Surgical technique
e. Experience of operator
f. Do not know

4. **What do you think is the most common early complication associated with dental implant?**

a. Permanent altered sensation
b. Implant failure
c. Unfavourable implant location compromising the prosthetic rehabilitation
d. Postoperative infection
e. Invasion of maxillary sinus
f. life-threatening hemorrhage
g. Do not know

5. **What do you think is the most common late complication associated with dental implant?**
a. Loss of prosthetic rehabilitation
b. Implant loss not resulting in the loss of the prosthetic rehabilitation
c. Massive bone loss related to implant failure
d. Do not know

6. **What do you think is the most common** mechanical **complication associated with dental implant?**
a. Fracture of implants
b. Fracture of screws
c. Fracture of abutments
d. Screws loosening
e. Abutment loosening
f. Fracture bridge frameworks
g. Do not know

7. **What do you think is the most common soft tissue complication associated with dental implant?**

a. Hemorrhage
b. Nerve injury
c. Tissue emphysema
d. Infections
e. Wound dehiscence
f. Do not know

8. **What do you think is the most common hard tissue complication associated with dental implant?**

a. Periapical implant pathosis
b. Mandibular jaw fracture
c. Lack of implant primary stability,
d. Inadvertent penetration into maxillary sinus or nasal fossa
e. Complications associated with sinus elevation
f. Do not know

9. **What do you think is the most common aesthetic complication associated with dental implant?**

a. Loss of interdental papilla
b. Gingival recession
c. Exposure of implant margin
d. Restoration too buccal or too palatal
e. Poor emergence profile
f. Chronic inflammation
g. Do not know

**10. What do you think is the most common reversible complication associated with dental implant?**

a. Intraoperative complications
b. Immediate/early postoperative complications
c. Late postoperative complications
d. Prosthetic-related (mechanical/biologic) complications
e. Aesthetic/soft tissue-related complications
f. Do not know
